# Preparation of Iron Ore Tailings-Based Superhydrophobic Coatings

**DOI:** 10.3390/ma15124235

**Published:** 2022-06-15

**Authors:** Zhiyuan Su, Qingguo Tang, Weiwei Zhao, Cong Liang, Qian Liu, Fei Wang, Xinhui Duan, Jinsheng Liang

**Affiliations:** 1 Institute of Power Source and Ecomaterials Science, Hebei University of Technology, Tianjin 300130, China; suzhiyuan970118@163.com (Z.S.); zw18653723581@163.com (W.Z.); liang1097@126.com (C.L.); liuqian_0327@163.com (Q.L.); wangfei@hebut.edu.cn (F.W.); dxh1191984@aliyun.com (X.D.); liangjinsheng@hebut.edu.cn (J.L.); 2 Key Laboratory of Special Function Materials for Ecological Environment and Information, Ministry of Education, Hebei University of Technology, Tianjin 300130, China

**Keywords:** iron ore tailings, superhydrophobic, wettability, chloroprene rubber solution

## Abstract

In this study, ball mill pretreated iron ore tailings were modified with tetraethoxysilane (TEOS) and hexadecyltrimethoxysilane (HDTMS) to obtain iron ore tailings/polysiloxane (IOT/POS) superhydrophobic powders, which were subsequently mixed with chloroprene rubber solution (CRS) to prepare durable superhydrophobic composite coatings. The effect of HDTMS amount and reaction time on the wettability of the superhydrophobic powder was investigated. The influence of the superhydrophobic powders concentration on the wettability of the composite coatings as well as the degree of damage of the superhydrophobicity of the composite coating was analyzed by using the sandpaper abrasion and tape peeling tests. Further, SEM and FTIR were used to analyze the formation mechanism of the IOT/POS superhydrophobic powders and coatings. The results showed for an HDTMS amount of 2.5 mmol and reaction time of 4 h, the contact angle of the IOT/POS powder was 157.3 ± 0.6°, whereas the slide angle was determined to be 5.9 ± 0.8°. For an IOT/POS powder content of 0.06 g/mL in CRS, the contact angle value of the superhydrophobic composite coating was 159.2 ± 0.5°, whereas the slide angle value was 5.5 ± 0.8°. The superhydrophobic composite coating still maintained the superhydrophobicity after the sandpaper abrasion and tape peeling tests, which indicated the iron ore tailings solid waste has the potential to prepare superhydrophobic coatings.

## 1. Introduction

The superhydrophobic coatings have attracted an increasing attention due to their promising applications in various fields, such as self-cleaning [1,2,3], anti-icing [4,5,6], corrosion resistance [7,8], and drag reduction [9,10]. The cooperation of appropriate micro/nano scale composite structure and the low-surface-energy modifier is the basic strategy for fabricating superhydrophobic coatings [11]. Based on this strategy, researchers used SiO_2_, TiO_2_, and carbon nanotubes as skeleton materials to produce the superhydrophobic powders or suspensions through hydrothermal synthesis, sol-gel synthesis, graft coating and other processes, followed by spraying or dipping to prepare superhydrophobic coatings [12,13,14,15]. Alternatively, aluminum, copper, and other bulk metal surfaces are employed as raw materials, followed by the corrosive dissolution, mechanical abrasion, and other processes to form a rough surface. Subsequently, spraying the nanoparticles modified with low surface energy modifier leads to the preparation of the superhydrophobic coatings [16,17,18,19]. However, the complicated and expensive preparation methods and the low stability of most of the reported superhydrophobic coatings limit their practical application. Thus, a few research studies have employed inexpensive nanostructured clay minerals as skeleton materials, along with their modification with silane coupling agents and introduced an adhesive into the coating to prepare superhydrophobic coatings. For instance, Li et al. [20] prepared a superhydrophobic coating with excellent anti-corrosion properties by spraying the water-based polyurethane and octadecyltrimethoxysilane-modified diatomite powders. Zhang et al. [21] prepared a superhydrophobic coating with excellent stability and transparency by spraying the organosilane/attapulgite nanocomposites. Chen et al. [22] used tetraethylorthosilicate and hexadecyltrimethoxysilane to modify sepiolite and incorporated it in an epoxy resin suspension to prepare superhydrophobic, mechanically strong and chemically stable coatings. However, high quality clay minerals are still scarce, and the price of beneficiation and purification is still high. In this respect, the use of industrial waste, which is similar in composition as the clay minerals, to prepare the superhydrophobic coating materials represents a functional strategy. Therefore, it is necessary to find lower-cost skeleton materials to prepare high-performance superhydrophobic coatings.

As a kind of bulk solid waste, coarse-grained iron ore tailings were widely used as sand and stone material for construction and produced glass ceramics [23,24], porous ceramsite [25], cement clinker [26]. However, the high value-added industrial utilization of microfine iron ore tailings has become an urgent problem that needs to be solved [27]. Because of the similar composition between iron ore tailings and clay, microfine iron ore tailings may be an ideal skeleton material for developing superhydrophobic coatings to further improve its application value. Therefore, this study focuses on the feasibility of iron ore tailings as superhydrophobic coating materials.

In addition, rubber materials have excellent high elasticity and wear resistance. Compared with epoxy and polyurethane adhesive, rubber adhesive can not only effectively improve the durability of superhydrophobic coatings but also provides enhanced elasticity to the coating, allowing the coating to better dissipate wear. Milionis et al. [28] first sprayed a liquid rubber adhesive (Plasti Dip™) on the aluminum substrate as a primer, then sprayed a polymer-MWCNTs mixed solution to enhance the thermal stability of the coating, and finally sprayed acrylonitrile buta-diene benzene. The mixed solution of ethylene rubber and hydrophobic fumed silica was used as the hydrophobic layer. After heat curing, a superhydrophobic coating was obtained. The strong adhesion from the binder made the coating resistant to 1700 linear wear cycles at 20.5 kPa, and the presence of multi-walled carbon nanotubes made it resistant to high temperatures of 42 °C. Wimalasiri et al. [29] first mixed polychloroprene adhesive and ethyl acetate to prepare adhesive solution, then the solution was sprayed on the surface of vulcanized natural rubber, and then superhydrophobic coating solution made of organosilicon sealant, organically modified silica aerogel and turpentine oil was sprayed. After thermal curing, superhydrophobic coating with good durability was prepared. Therefore, using rubber as adhesive is an important measure to improve the durability of superhydrophobic coatings.

Based on the above discussion, in this study, the industrial waste iron ore tailings after ball milling are used as a skeleton material, followed by modifying with TEOS and HDTMS to obtain superhydrophobic powders, and using chloroprene rubber solution as adhesive, to prepare the durable superhydrophobic coatings.

## 2. Experimental Section

### 2.1. Materials

The main mineral composition of the IOT was made up by silicate minerals. IOT (supplied by Jianlong Mining Co. Ltd., Hebei, China) was composed of SiO_2_ (39.0%), Fe_2_O_3_ (19.1%), MgO (11.2%), CaO (14.2%), Al_2_O_3_ (9.87%), TiO_2_ (1.65%), K_2_O (1.53%), and P_2_O_5_ (1.30%), as determined by X-ray fluorescence spectrometry (Rigaku, cZSX Primus2, Japan). Chloroprene rubber (NEC, DCR36, Tokyo, Japan) was produced. Tetraethoxysilane (TEOS), dichloromethane (CH_2_Cl_2_), diiodomethane (CH_2_I_2_), absolute ethanol, aqueous ammonia (NH_3_H_2_O), and hexadecyltrimethoxysilane (HDTMS) were purchased from Tianjin Kmart Chemical Technology Co. Ltd. All chemical reagents were used without further purification.

### 2.2. Preparation of IOT/POS Powders

IOT was ball milled in order to refine it and increase its specific surface area, which is beneficial for the construction of the micro/nanoscale rough structure and modification reaction. The diameters D_50_ and D_90_ were changed before and after ball milling, from 2.901 μm to 1.231 μm, and 21.304 μm to 2.816 μm, respectively.

The IOT/POS powders were prepared via NH_3_H_2_O-catalyzed hydrolytic condensation of HDTMS and TEOS in the presence of IOT. First, 1.0 g IOT was added to a 50 mL flat-bottomed flask containing 10 mL deionized water and 25 mL absolute ethanol (The mentioned IOT here and later represents the iron ore tailings after ball milling pretreatment). Afterwards, the solution was ultrasonically dispersed for 30 min, followed by stirring for 10 min. Subsequently, 2.0 mL ammonia, 6.5 mmol TEOS, and different amounts of HDTMS were added to the suspension and mixed till a uniform suspension was obtained. The precipitate of the resulting suspension was washed three times with ethanol by centrifugation at 5000 rpm for 10 min. Afterwards, the product was dried overnight in an oven at 60 °C to finally obtain the IOT/POS powders.

### 2.3. Preparation of Composite Coatings

Total of 0.3 g chloroprene rubber was added to a reactor containing 80 mL dichloromethane. The reactor was placed in an oven at 150 °C for 10 h to fully dissolve the chloroprene rubber in order to obtain CRS. Different amounts of IOT/POS powders were added to 50 mL CRS and stirred to mix well. Afterwards, ultrasonic dispersion of the suspensions was carried out for 30 min, followed by spraying of the suspensions on the copper sheet. Finally, curing at 120 °C for 2 h led to the generation of the composite coatings.

### 2.4. Durability Test of Composite Coatings

**Figure 1:**
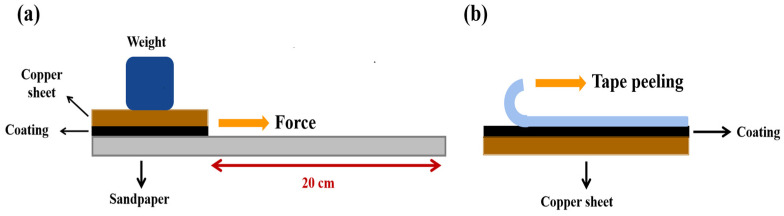
The schematic of sandpaper abrasion and tape peeling tests. (**a**) Sandpaper abrasion test (**b**) tape peeling test.

Many tests have been developed to verify the durability of coatings [30,31]. Among them, sandpaper abrasion and tape peeling tests are widely used, thus we chose these two tests to evaluate the durability of superhydrophobic coatings. During the sandpaper abrasion test, a 25 × 35 × 5 mm composite coating was moved a distance of 20 cm at a speed of 3 cm/s on the surface of sandpaper (1000 Cw) under a load weight of 100 g (considered as one cycle). The CA and SA values were recorded after every ten cycles. In the tape peeling test, the 3M tape was applied on the composite coating and pressed to ensure that the entire adhesive surface was in contact with the surface of the composite coatings. The CA and SA values were determined after every three tape removals. The schematic of sandpaper abrasion and tape peeling tests is shown in the Figure 1.

### 2.5. Characterizations

The contact angle (CA) and slide angle (SA) were measured using the Contact Angle System OCA20 (Dataphysics, Filedrstadt, Germany) equipped with a tilting table by placing 5 μL deionized water at five different positions on the sample surface and recording an average value. Tilting angle of the table was adjustable (0–70°) and allowed the subsequent measurement of the SA at the same position on the sample. The microstructure of the samples was observed by scanning electron microscopy (SEM, Quanta 450 FEG, Hongkong, China) operated at 10 kV. The functional groups of the samples were examined by using Fourier transform infrared spectroscopy (FTIR, Bruker V80, Karlsruhe, Germany). Optical profilometer (Bruker, CountorGTK, Karlsruhe, Germany) are used to measure the surface roughness of the samples.

## 3. Results and Discussion

### 3.1. Effect of the HDTMS Amount on the Wettability of IOT/POS

**Figure 2:**
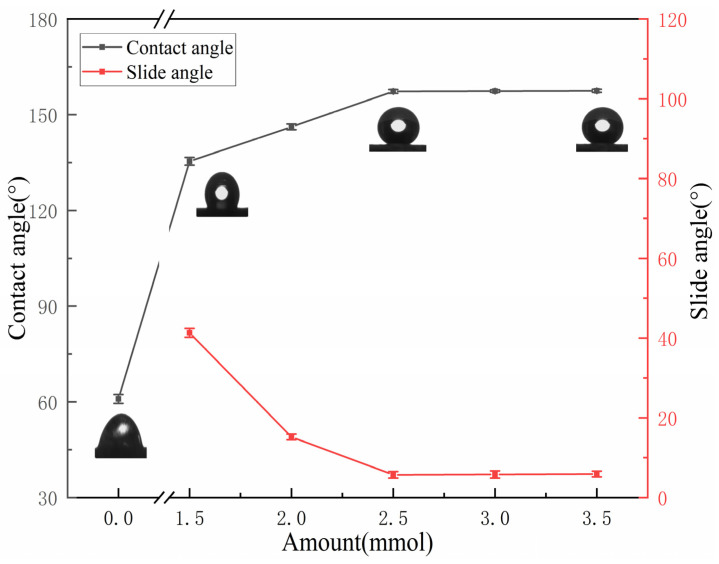
Variation of the wettability of the IOT/POS powders as a function of the HDTMS amount.

The effect of the HDTMS amount on the wettability of IOT/POS is shown in Figure 2 (TEOS amount was 6.5 mmol and reaction time was set to 4 h). In the absence of HDTMS, the CA value of the modified IOT powder was 60.9 ± 1.4°. On increasing the HDTMS amount, the CA values of the powder presented an initial increasing trend, followed by stabilization, and the SA values gradually decreased and eventually became stable. On adding 2.5 mmol HDTMS, the CA value increased to 157.3 ± 0.6°, whereas the SA decreased to 5.9 ± 0.8°. On further increasing the amount of HDTMS, the change in the CA and SA values of the powder tended to be slow. Therefore, an amount of 2.5 mmol HDTMS was selected for the subsequent studies.

### 3.2. Effect of the Reaction Time on Wettability of IOT/POS

**Figure 3:**
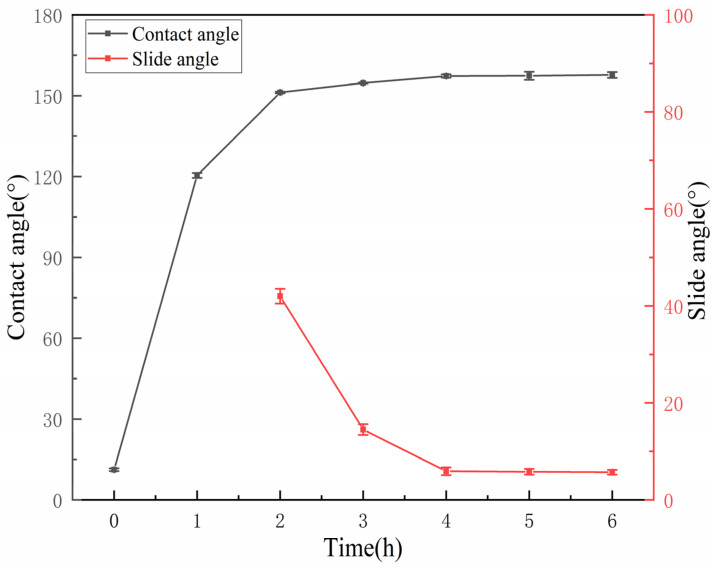
Variation of the wettability of the IOT/POS powders with reaction time.

The influence of reaction time on the wettability of the IOT/POS powders is shown in Figure 3 (TEOS amount was 6.5 mmol and HDTMS amount was 2.5 mmol). On increasing the reaction time, the CA value of the powder first increased and subsequently stabilized, whereas the SA value first declined and gradually tended to be stable. The CA and SA values reached 157.3 ± 0.6° and 5.9 ± 0.8° after 4 h. The wettability of the powder tended to be stable with the reaction time. As the reaction time increased, the silane amount was also enhanced through hydrolysis and condensation, thus, resulting in a high degree of IOT surface coating modification and enhanced hydrophobic characteristic of the generated powder. After 4 h, the coating modification of IOT was basically complete, and further extending the reaction time had a little effect on the wettability of the powder. Considering the wettability trend and time, 4 h was chosen as the optimal reaction time for subsequent experiments.

### 3.3. Effect of IOT/POS Powder Concentration on Wettability of Composite Coatings

**Figure 4:**
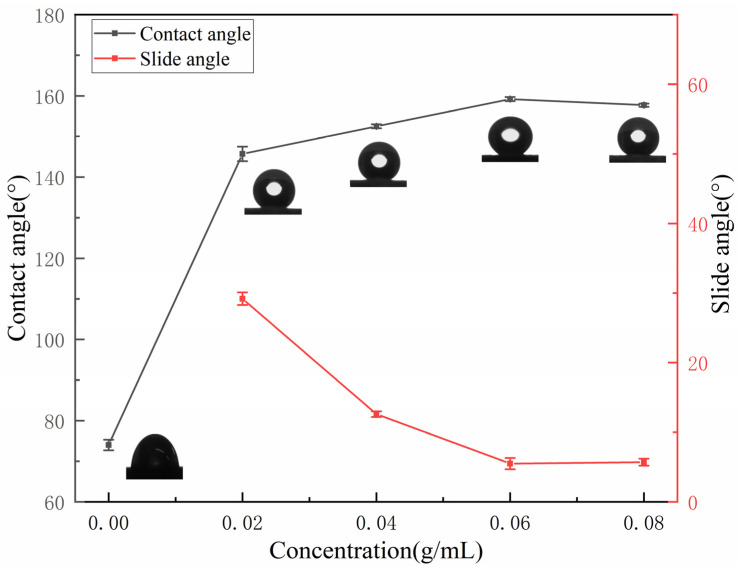
Effect of IOT/POS powder concentration on wettability of composite coatings.

Figure 4 shows the relationship between the concentration of IOT/POS powders and wettability of the superhydrophobic coatings. The CA value of pure CRS coating was 74 ± 1.3°. The value was observed to be significantly increased, whereas the SA value decreased, with the increase of IOT/POS powders concentration. For a concentration of 0.06 g/mL, the CA value of the coating was 159.2 ± 0.5°, whereas the SA value was determined to be 5.5 ± 0.8°. However, on further increasing the concentration of IOT/POS powders, the CA value of the coatings was reduced, whereas the SA value was increased. Therefore, the composite coatings prepared with 0.06 g/mL IOT/POS powders were selected for subsequent studies.

### 3.4. Durability Testing of Composite Coatings

One of the challenges for practical application is the inferior durability of the superhydrophobic surfaces. Most artificially fabricated superhydrophobic surfaces swiftly lose their superhydrophobicity when exposed to the harsh mechanical conditions [32,33]. Thus, sandpaper abrasion and tape peeling tests were carried out to analyze the durability of the superhydrophobic composite coatings.

**Figure 5:**
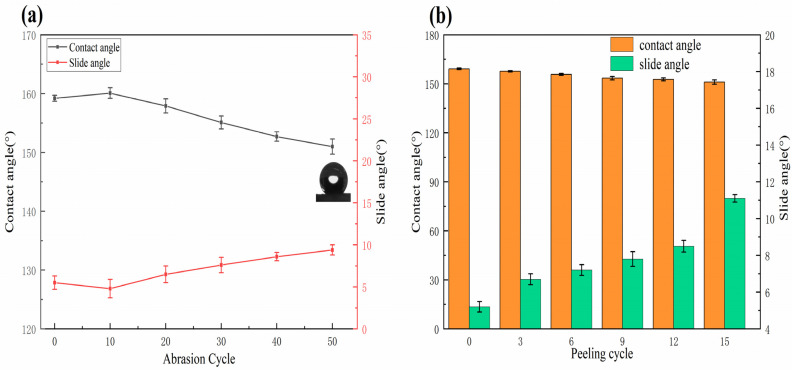
(**a**) Variation of superhydrophobicity of the composite coating during the sand abrasion test and (**b**) the tape peeling test.

The superhydrophobic behavior of the composite coatings as the number of abrasion cycle is shown in Figure 5a. Owing to the sandpaper abrasion, the roughness of the coating increased in the beginning, leading to a slight increment in the CA value. As the abrasion cycles reached 50, the CA value of the composite coating reduced to 151.1 ± 1.3°, whereas the SA value increased to 9.4 ± 0.7°. Figure 5b shows the variation of the superhydrophobicity of the composite coating during the tape peeling test. After 15 cycles, the CA value of the composite coating decreased to 151.1 ± 1.3°, whereas the SA value increased to 11.0 ± 0.5°. These results indicated that the superhydrophobic composite coatings exhibited an excellent durability.

### 3.5. Analysis of Surface Energy and Surface Topography Changes

The micro/nano scale rough structure and low surface energy are the two key factors for obtaining a superhydrophobic surface [34,35]. In order to explore the changes in the surface energy of IOT before and after modification as well as after adding CRS to the coating, the surface energy of IOT, IOT/POS, and composite coating was calculated according to Owens’ theory, as shown in Equations (1)–(3):

(1) $$\gamma_{s}=\gamma_{s}^{p}+\gamma_{s}^{d}$$

(2) $$\gamma_{l}=\gamma_{l}^{p}+\gamma_{l}^{d}$$

(3) $$(\cos\theta +1)\gamma_{l}=2(\gamma_{s}^{d}\gamma_{l}^{d}{)}^{{}^{1/2}}+2(\gamma_{s}^{p}\gamma_{l}^{p}{)}^{1/2}$$

where, γ_s_ is the surface energy of the solid; $\gamma_{s}^{p}$ and $\gamma_{s}^{d}$ are polar and nonpolar sections of γ_s_, respectively; γ_l_ is the surface energy of the liquid, $\gamma_{l}^{p}$ and $\gamma_{l}^{d}$ are polar and nonpolar sections of γ_l_, respectively; and θ is the CA value of the test liquids. Choosing diiodomethane and water as the typical liquids (Table 1), the surface energy of IOT, IOT/POS and composite coating was calculated as 80.1 mN/m, 35.4 mN/m, and 30.7 mN/m, respectively. Thus, the surface energy of the modified IOT was obviously reduced, and the addition of CRS further reduced the surface energy.

**Figure 6:**
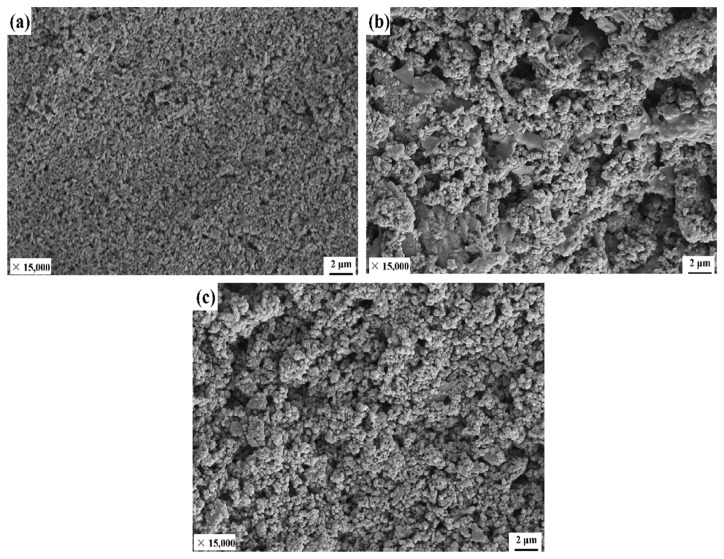
SEM images of IOT after ball milling (**a**) and SEM images of IOT/POS for a HDTMS amount of (**b**) 1.5 mmol and (**c**) 2.5 mmol.

It can be observed from the previous experiments that changing the HDTMS amount affects the wetting performance of the IOT/POS powders, attributed to the changes in the surface microstructure. Figure 6a is the SEM image of IOT prior to modification. IOT after ball milling was observed to be broken into multi-shaped particles. Figure 6b,c presents the SEM images of modified iron tailings after the addition of HDTMS (1.5 mmol and 2.5 mmol, respectively). As observed, the surface of IOT became rough after modification. This was attributed to the hydrolysis and condensation reaction of TEOS under alkaline conditions, leading to the generation of SiO_2_ nanoparticles. These nanoparticles were irregularly stacked on the surface of IOT at a micrometer scale to form the micro/nano composite structure. For a HDTMS amount of 2.5 mmol, the SiO_2_ nanoparticles were more uniformly coated on the surface of IOT. This indicated that a high amount of HDTMS could induce the SiO_2_ nanoparticles to aggregate on the surface of IOT, imparting the IOT/POS powders a high surface roughness. The observed phenomenon could be attributed to the superior wettability.

**Figure 7:**
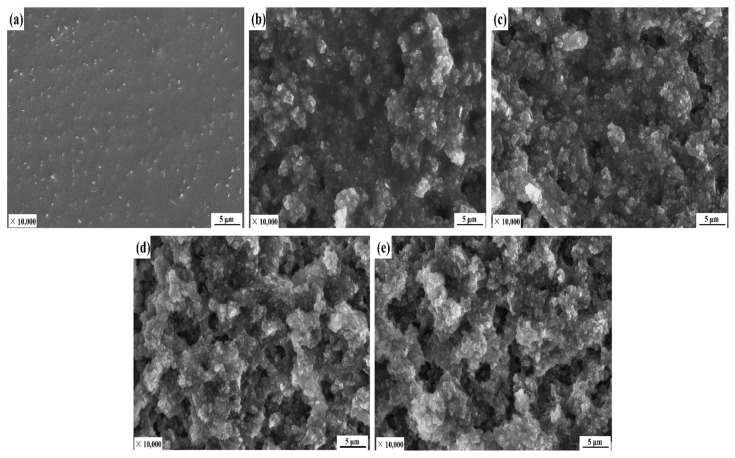
SEM images of composite coatings prepared with different concentration of IOT/POS powders: (**a**) 0 g/mL, (**b**) 0.02 g/mL, (**c**) 0.04 g/mL, (**d**) 0.06 g/mL, and (**e**) 0.08 g/mL.

The previous experiments also prove that the concentration of IOT/POS superhydrophobic powders has a significant effect on the wettability of the composite coatings. This is also related to the changes in the surface microscopic topography. Figure 7 presents the SEM images of the composite coatings prepared using different addition concentration of IOT/POS powders. As shown in Figure 6a, the surface of the CRS coating was smooth with no rough structure, thus, indicating that the rough surface required for the superhydrophobic coating could not be provided by spraying the CRS reagent solely. However, as can be seen from Figure 7b–e, the addition of IOT/POS powder directly led to the formation of the micro-nanoscale composite structure on the surface. The micro-nanoscale composite structure was led by the cross-linking of CRS and IOT/POS powders during the curing process. At a high concentration of the IOT/POS powder, as shown in Figure 7d,e, the IOT/POS particles were observed to be prone to agglomeration, thus, producing holes of different sizes and depths on the coating surface. The unique micro-nano structure and holes can capture a large amount of air and reduce the solid–liquid contact area, thus, improving the wetting performance of the coating. At a low concentration of the IOT/POS powders, as shown in Figure 6b,c, a majority of the IOT/POS particles were completely wrapped by CRS, and the coating surface had only a few holes. Further, the ability to capture air lessened, thereby resulting in a smaller contact angle of the coating. However, the difference between Figure 7d,e was not obvious.

**Figure 8:**
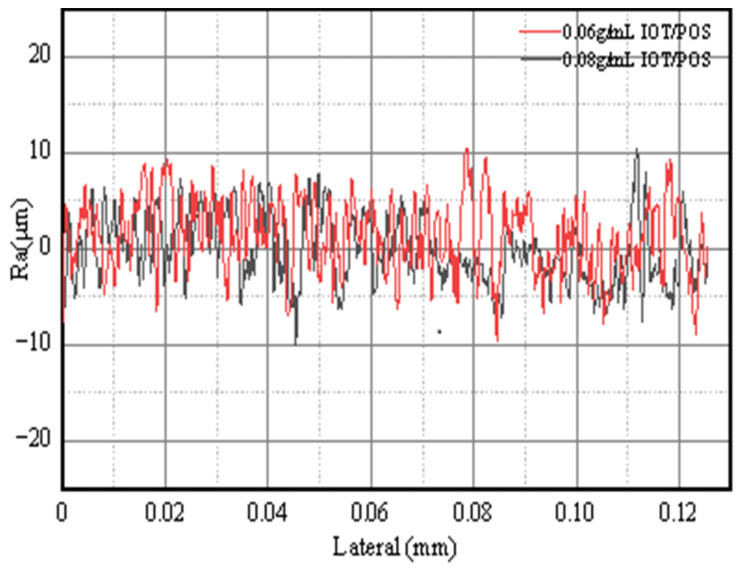
Profilometry results for the composite superhydrophobic coatings with 0.06 g/mL and 0.08 g/mL IOT/POS powders.

In order to further explore the reasons for the change in wettability, the surface roughness of the composite coatings with IOT/POS concentration of 0.06 g/mL and 0.08 g/mL were measured by optical profilometer. The result is shown in Figure 8.

Results showed that when the IOT/POS concentration is 0.06 g/mL, the average roughness value of the coating is 3.471 μm. The average roughness value of the composite coating with an IOT/POS concentration of 0.08 g/mL is 2.619 μm. This indicates that the increased concentration of IOT/POS powders leads to a decrease in the average surface roughness of the coating, thereby resulting in a decline in wettability of the coating.

**Figure 9:**
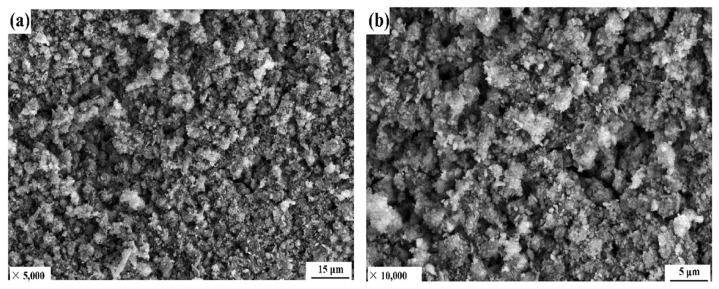
Low and high-magnification SEM images of the composite superhydrophobic coating with 0.06 g/mL IOT/POS powders after 1000 cm wear distance abrasion (**a**,**b**).

To further explore the reasons for the excellent durability of the coating, the morphology of the coating surface after abrasion was observed with SEM. Figure 9a,b presents the low and high magnification SEM images of the composite coating surface after 1000 cm wear distance, respectively. It can be seen that the macrostructure was partially destroyed from the low magnification SEM image. The high magnification SEM image of the coating surface demonstrated no obvious change in the micro/nano structure, which was the key reason explaining the retention of superhydrophobicity of the composite coatings after abrasion. It indicated that the cured CRS contributed toward fixing the IOT/POS particles, thus, preventing the microstructure of the coating surface from being damaged during the friction process. In addition, strong adhesion between the coating and substrate was also generated, thus, the coating did not fall off during the abrasion processes easily.

### 3.6. FT-IR Analysis

**Figure 10:**
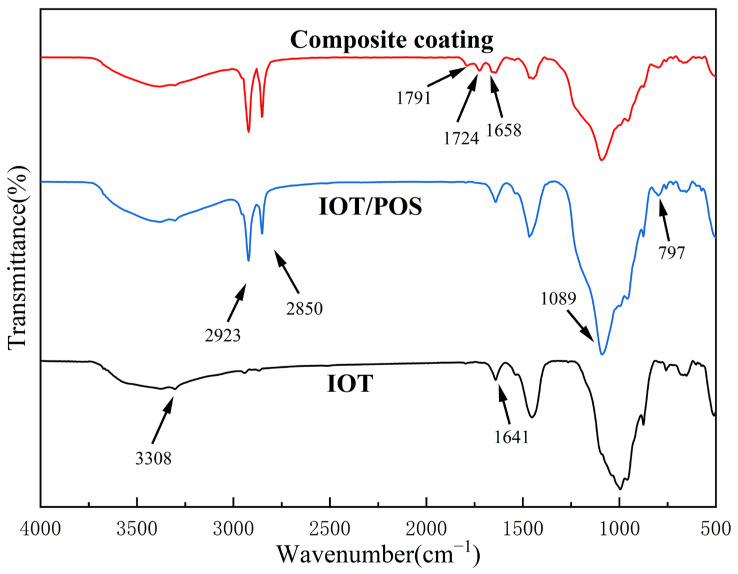
FTIR spectrum of IOT, IOT/POS and composite coating with 0.06 g/mL IOT/POS powders.

The surface wetting behavior is not only affected by the surface microstructure, but also by the surface functional groups. The FTIR spectra, as shown in Figure 10, were used to analyze the changes in the functional groups. For the raw IOT, characteristic peaks were observed at nearly 3308 cm^−^^1^ and 1604 cm^−^^1^ attributed to the O-H bond. As for the IOT/POS powders, new peaks at 2923 cm^−1^ and 2850 cm^−1^ were attributed to the -CH_3_ and -CH_2_ groups, respectively, which originated from the HDTMS modifier. In addition, the peaks at 1089 cm^−^^1^ and 797 cm^−^^1^ were attributed to the Si-O-Si bond. The appearance of the new peaks indicated that IOT was successfully modified with POS. Moreover, it was noted from the FTIR spectra of the superhydrophobic coating that the C=C stretching vibration peak from chloroprene rubber appeared at 1658 cm^−^^1^. Further, the characteristic peaks of the C=O group appeared at 1791 cm^−^^1^ and 1724 cm^−^^1^, which was due to the reason that the (CH=C)-Cl structure of CRS combined with oxygen to produce oxygen-containing groups during the curing process. These results indicated that the IOT/POS powders could be effectively combined with CRS. In addition, grafting of CH_3_ and CH_2_ from the long-chain alkyl groups in HDTMS endowed the IOT/POS powders and superhydrophobic composite coatings with low surface energy.

### 3.7. Self-Cleaning Test

**Figure 11:**
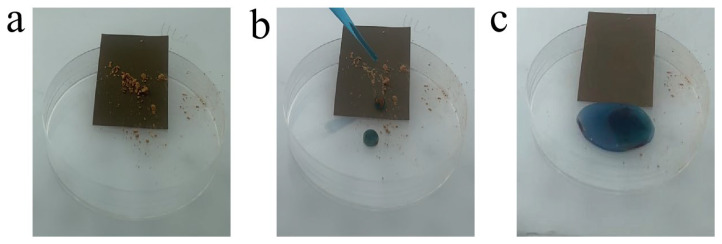
The process of composite coating self-cleaning (**a**–**c**).

Self-cleaning is a unique feature for superhydrophobic surfaces that automatically cleans surface dirt with water. In order to investigate the self-cleaning properties of superhydrophobic composite coating that we obtained, we deposited red attapulgite as a contaminant on the coating surface, with a slight inclination angle (Figure 11a). When water droplets dyed by methyl blue rolled down from the coating surface, it was found that the water droplets retained the round shape and washed away the contaminants (Figure 11b). This is related to the low surface energy of the coating. After rinsing with water, the surface was clean (Figure 11c). The above results demonstrate that the composite coatings we obtained have good self-cleaning property.

## 4. Conclusions

In conclusion, a low-cost method was developed in this study to prepare durable superhydrophobic composite coatings, which were facilely designed by spraying a mixed suspension of iron ore tailings/polysiloxane (IOT/POS) and chloroprene rubber solution (CRS). The amount of HDTMS and reaction time exhibited a significant influence on the wettability of IOT/POS powders. Further, the concentration of IOT/POS superhydrophobic powders also had a marked effect on the wettability of the composite coating. Owing to the irregular stacking of the SiO_2_ nanoparticles on the surface of IOT with a micrometer scale, a micro/nano composite structure was generated. Grafting of long-chain alkylsilane of HDTMS endowed IOT/POS and composite coating with a low surface energy. CRS not only contributed toward fixing the IOT/POS particles, but also formed a strong adhesion between the coating and substrate. Therefore, the developed superhydrophobic composite coatings displayed a high water CA, low SA, and excellent durability and self-cleaning property. Further, this study also provides the basis for the preparation of superhydrophobic coatings from other solid wastes.

## Figures and Tables

**Table 1:** Polar and nonpolar sections of typical liquids.

Typical Liquids	$\gamma_{l}^{d}$ (mJ/m^2^)	$\gamma_{l}^{p}$ (mJ/m^2^)	γ_l_ (mJ/m^2^)
diiodomethane	50.8	0.0	50.8
water	21.8	51.0	72.8

## Data Availability

Not applicable.
